# Fibrillin-1 mutation contributes to Marfan syndrome by inhibiting Cav1.2-mediated cell proliferation in vascular smooth muscle cells

**DOI:** 10.1080/19336950.2023.2192377

**Published:** 2023-03-27

**Authors:** Wenfeng Lin, Jiaqi Xiong, Yefan Jiang, Hao Liu, Jinhui Bian, Juejin Wang, Yongfeng Shao, Buqing Ni

**Affiliations:** a Department of Cardiovascular Surgery, The First Affiliated Hospital of Nanjing Medical University, Nanjing, Jiangsu, China; b Key Laboratory of Cardiovascular Disease and Molecular Intervention, Department of Physiology, Nanjing Medical University, Nanjing, Jiangsu, China

**Keywords:** Marfan Syndrome, Cav1.2, FBN1, cell proliferation, cell cycle

## Abstract

Marfan syndrome (MFS) is an autosomal dominant connective tissue disorder caused by mutation in fibrillin-1 (FBN1). However, the molecular mechanism underlying MFS remains poorly understood. The study aimed to explore how the L-type calcium channel (Ca_V_1.2) modulates disease progression of MFS and to identify a potential effective target for attenuating MFS. KEGG enrichment analysis showed that the calcium signaling pathway gene set was significantly enriched. We demonstrated that FBN1 deficiency exhibited inhibition on both the expression of Cav1.2 and proliferation of vascular smooth muscle cells (VSMCs). Then, we examined whether FBN1 mediates Cav1.2 via regulating TGF-β1. Higher levels of TGF-β1 were observed in the serum and aortic tissues from patients with MFS. TGF-β1 modulated Cav1.2 expression in a concentration-dependent manner. We evaluated the role of Cav1.2 in MFS by small interfering RNA and Cav1.2 agonist Bay K8644. The effect of Cav1.2 on cell proliferation was dependent on c-Fos activity. These results demonstrated FBN1 deficiency decreased the expression levels of Cav1.2 via regulation of TGF-β1, and downregulation of Cav1.2 inhibited cell proliferation of human aortic smooth muscle cells (HASMCs) in MFS patients. These findings suggest that Cav1.2 may be an appealing therapeutic target for MFS.

## Introduction

Marfan syndrome (MFS) is an autosomal dominant disorder with an estimated prevalence of one in 5000 individuals [1]. MFS accounts for approximately 5% of all cases of aortic dissection, a severe disease with high morbidity and mortality [2]. MFS is known to be caused by mutations in *FBN1*, which encodes the elastic microfibril protein FBN1 [3,4]. Some studies have shown that FBN1 interacts with latent TGF-β binding proteins (LTBP) and controls TGF-β1 bioavailability [5]. Increased activation of TGF-β1 is due to microfiber breakage caused by FBN1 dysfunction. The development of aortic aneurysms in MFS patients is closely related to TGF-β1 levels [6,7].

HASMCs are the predominant cell type in the tunica media of blood vessels. Studies have reported that rapid regeneration of HASMCs is essential for maintaining arterial function, and vessel regeneration is mediated by the proliferative expansion of preexisting HASMCs [8]. Furthermore, HASMCs proliferation is reportedly impaired in MFS patients [9].

Cav1.2, one L-type voltage-gated calcium channel, plays a crucial role in regulating various cellular processes including neuronal transmission, muscle contraction, hormone secretion, and gene expression [10–13]. It is now generally accepted that calcium influx via Cav1.2 is the main source of intracellular calcium in VSMCs [14]. Ca^2+^ operates as a secondary messenger to activate nuclear calcium-dependent enzymes and transcription factors, such as NFAT3, GATA4, and c-Fos [15–17]. The transcription factors modulated by Ca^2+^ are critical for cell survival, plasticity, proliferation, and differentiation [18]. As immediate early genes (IEGs), c-Fos can control the entry of G0 resting cells into the cell cycle and promote cell proliferation. After growth factor stimulation, c-Fos transcriptional activity occurs within minutes and precedes that of other IEGs [19]. Additionally, Cav1.2 is associated with the proliferation of bladder SMCs, osteoblasts, and glial cells [18,20,21]. The proliferation of HASMCs is beneficial to the stability of vascular function. However, there is no reports of Cav1.2 regulating the proliferation of HASMCs, and the relationship between FBN1 and Cav1.2 remains ill-defined.

In the present study, we elucidated a novel FBN1/Cav1.2 pathway in MFS. This finding provides the first demonstration that FBN1 deficiency leads to the decline of Cav1.2 expression due to TGF-β1 over-expression, which inhibits the proliferation of HASMCs in MFS patients (MFS-HASMCs).

## Materials and methods

### Human tissue and data collection

The required approval was obtained for the collection of human tissue samples. All studies involving humans complied with the Declaration of Helsinki and were approved by the Ethics Committee of the First Affiliated Hospital of Nanjing Medical University (IRB number:2019-SR-067; date of IRB approval: 12 March 2019). Written informed consent was obtained from all participants before surgery. Control ascending aortic specimens (*N* = 11) were collected from patients with acute aortic dissection at the First Affiliated Hospital of Nanjing Medical University. Experimental specimens (*N* = 5) were collected from age-matched MFS patients who underwent aortic aneurysm repair. MFS was diagnosed in all patients based on Ghent nosology. Samples were taken from the dilated ascending aneurysms of patients. The aortic tissues were kept in DMEM during laboratory delivery.

### Cell culture

HASMCs were isolated from control donors (control-HASMCs) and Marfan donors (MFS-HASMCs). Tunica media was cut into 1–2 mm^3^ cubes and transferred to *T*-25 culture flasks. The aortic media small cubes were gently covered with 10 mL DMEM after adhesion at 37°C for 14 h. The cells were cultured in a humidified 5% CO_2_ environment at 37°C. Explants were left undisturbed for 5 days unless the medium turned yellow, and three quarters of the medium was changed every 3 days. After 1–2 weeks, HASMCs migrated from explants. After removing the explants from the flask surface, cells were trypsinized and used as P2 stage cells. Cells were used between passage number 4 and 8 (P4–P8) in all experiments. Cell morphology is showed in Supplemental Figure 1.

### Western blot analysis

Tissue and cell proteins were extracted with RIPA buffer containing protease inhibitors (New Cell & Molecular Biotech Co., Ltd, Jiangsu, China). Proteins were separated on 6% SDS-PAGE gel and transferred to PVDF membranes. GAPDH or Na+/K+ ATPase was used as an internal control. The bands were visualized with an ECL reagent (Vazyme Biotech Co., Ltd, Jiangsu, China). Image J (National Institutes of Health, New York, USA) was used to quantify the blots. The antibodies used are listed in Supplemental Table 1. Original blots are shown in the source data.

### Quantitative real-time PCR

Total RNA was extracted from HASMCs using TRIzol reagent (Ambion, Texas, USA) according to the manufacturer’s instructions. A TaKaRa RNA PCR kit (TaKaRa, Dalian, China) was used for reverse transcription. Real-time PCR was performed with SYBR Green (Vazyme), and data were collected using QuantStudio Real-Time PCR Software (Thermo). Relative quantification was performed using the 2^ΔΔCt^ method with GAPDH as a reference. Primer sequences used are listed in Supplemental Table 2.

### TGF-β1 ELISA

At room temperature, Marfan blood and healthy blood were coagulated for 1 h in serum tubes. The samples were spun in a centrifuge (75004524, Thermo Fisher, Massachusetts, USA) at 10,956 × g for 10 min. Serum was aliquoted and frozen at −80°C until analysis. The levels of TGF-β1 in the culture supernatant were determined using a Human TGF-β1 ELISA Kit (PT880, Beyotime Biotechnology, Shanghai, China) as directed by the manufacturer. The samples were treated with the activation reagent (1 N HCl) for 10 min at 25°C, followed by addition of neutralization reagent (1.2 N NaOH). Treated samples were incubated for 2 h at 25°C on ELISA plates coated with capture antibodies. Following three washes, samples were incubated for 1 h at 25°C with the detection antibody. ELISA plates were washed and incubated with HRP-conjugated secondary antibodies for 40 min at 25°C, followed by incubation for 20 min at 37°C with substrate solution. At 450 nm, optical density in each well was measured by Multiskan™ FC microplate reader (51119180ET, Thermo Fisher, Massachusetts, USA). Recombinant human TGF-β1 was used to prepare a standard curve.

### Immunofluorescence

ASMCs were grown on 24-well plates to 60–70% confluency. The cells were rinsed with PBS, fixed with pre-cooled absolute ethanol for 20 min, then rinsed three times with PBS. The cells were permeabilized and blocked with 0.3% Triton X-100 and 5% BSA Albumin Fraction V (BS114-100 g, Biosharp, Anhui, China) in PBS for 1 h at 25°C, then rinsed three times with PBS. The cells were incubated with primary antibody at 4°C overnight, then rinsed three times with PBS. The secondary antibody was added, and the cells were incubated at 25°C in the dark for 40 min, then rinsed three times with PBS. Cells were mounted with DAPI Fluoromount-G® (SouthernBiotech, Alabama, USA) to stain nuclei. Images were acquired by a Thunder DMi8 Fluorescence Microscope and imported into Adobe Photoshop CC 2019. The antibodies used are listed in Supplemental Table 1.

### Cell proliferation assay

HASMCs (3 × 10^3^) were cultured in a 96-well plate in DMEM with 10% fetal bovine serum (FBS) serum for 48 h. Cell proliferation was measured using Cell Counting Kit-8 (CCK8, Apex BIO Technology LLC, Texas, USA) according to the manufacturer’s instructions. DMEM was used as the control. CCK8 reagent (10 μL) was added to each well, followed by incubation for 2 h at 37°C. The absorbance at 450 nm was measured using a Multiskan™ FC microplate reader (51119180ET, Thermo Fisher, USA).

### Cell transfection

For siRNA transfection, HASMCs were plated in 6-well microtiter plates. HASMCs were transfected with control siRNA or a specific siRNA at a final concentration of 10 nmol/L using Lipofectamine 3000 (Invitrogen, California, USA) when HASMCs reached 80% confluence. After 24 h of transfection with Opti-MEM, the medium was replaced with DMEM containing 10% FBS. The siRNAs used are listed in Supplemental Table 3.

### RNA-seq analysis

Differences in transcript levels between Marfan patients and control donors were downloaded from the GEO database (GEO: GSE78833) which is publicly accessible at https://www.ncbi.nlm.nih.gov/geo. Differences in gene expression were analyzed by the Enrichr software (http://amp.pharm.mssm.edu/Enrichr/).

### Determination of intracellular calcium concentration

Pluronic F-127 was dissolved in DMSO at 0.2 mg/mL. Fluo −4 AM was then dissolved in 20% pluronic F-127 solution to 5 mM Fluo-4 AM solution (MX4540, MKBio, Shanghai, China). 5 mM Fluo-4 AM was diluted with DMEM without phenol red to a final concentration of 5 μM. HASMCs were washed 3 times in PBS, incubated with 5 μM Fluo-4 AM for 30 min and washed 3 times in DMEM without phenol red. HASMCs were then stimulated with 100 nM Cav1.2 agonist. Videos were taken with a Thunder Imaging System (Leica Microsystems), and the fluorescence intensity was analyzed using imageJ.

### Statistical analysis

Data analysis was performed using GraphPad Prism 9.4.1 (GraphPad Software, La Jolla, CA, USA). For cellular experiments, at least three repetitions were performed. Data are expressed as mean ± standard deviation. Differences were analyzed by the unpaired t-test or one-way analysis of variance. *P* < 0.05 was deemed to indicate statistical significance.

## Results

### Expression of Cav1.2 is decreased in MFS

**Figure 1.**
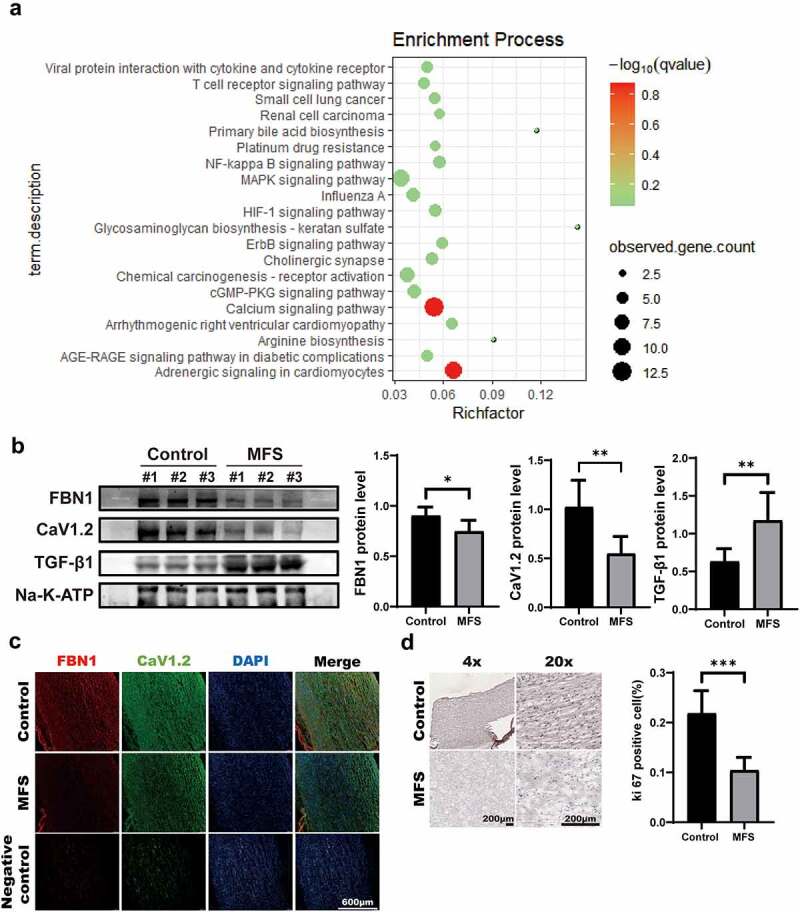
**The levels of Cav1.2 and cell proliferation in the ascending aortic tissues of MFS patients are lower compared with control donors. (a)** Expression differences between control-HASMCs and MFS-HASMCs. **(b)** Western blot and quantitative analysis of FBN1, Cav1.2 and TGF-β1 protein levels in the ascending aorta of control donors and MFS patients. **(c)** Representative images of immunofluorescent staining for FBN1 and Cav1.2 in the ascending aorta of control donors and MFS patients. Scale bar = 600 μm. **(d)** Representative images and quantitative analysis of ki67 immunohistochemical staining in the ascending aorta of control donors (*n* = 5) and MFS patients (*n* = 5). Scale bar = 200 μm. Data are expressed as the mean ± SD. *p < 0.05, **p < 0.01, ***p < 0.001. Source data are provided as a Source Data file.

To explore the molecular mechanism of MFS, we determined the differences in mRNAs between control-HASMCs and MFS-HASMCs by analyzing the GSE78833 dataset downloaded from the GEO database. The analysis identified significant enrichment for the calcium signaling pathway gene set in pathophysiological processes of MFS (Figure 1a). Voltage-gated calcium channel is determined by the main subunit α_1_ (13, 26). The mRNA levels of calcium channel in HASMCs with or without MFS were detected by analyzing the GSE78833 dataset. This data indicated that only two α1 subunits were changed (Supplemental Table 4). The *CACNA1C* (calcium voltage-gated channel subunit alpha1 C) was involved in the initiation of Cav1.2 formation, and the *CACNA1G* (calcium voltage-gated channel subunit alpha1 G) was thought to participate in the Cav3.1 formation. The mRNA levels for the indicated genes in the ascending aortic specimens of control donors and MFS patients were compared using quantitative real-time PCR (qRT-PCR). Notably, Cav1.2 transcript levels were downregulated in the ascending aorta of MFS patients (Supplemental Figure 2a). No statistically significant differences in Cav3.1 transcript levels and protein levels were observed (Supplemental Figure 2, a and b). We next compared the differences in Cav1.2 protein levels between Marfan aortic tissues and control tissues. Western blot analysis showed that Cav1.2 expression was significantly reduced in ascending aortic specimens in the three patients with MFS (Figure 1b). Furthermore, immunofluorescence illustrated that Cav1.2 and FBN1 were expressed at a lower level and were unevenly distributed compared to their expression and distribution in the control tissues (Figure 1c). Subsequent immunohistochemical staining of Ki67 for cell proliferation was carried out on the ascending aorta of control donors and MFS patients. Cell proliferation was significantly inhibited by MFS (Figure 1d). These data indicate that both Cav1.2 expression and cell proliferation are lower in tissues from donors with MFS.

**Figure 2.**
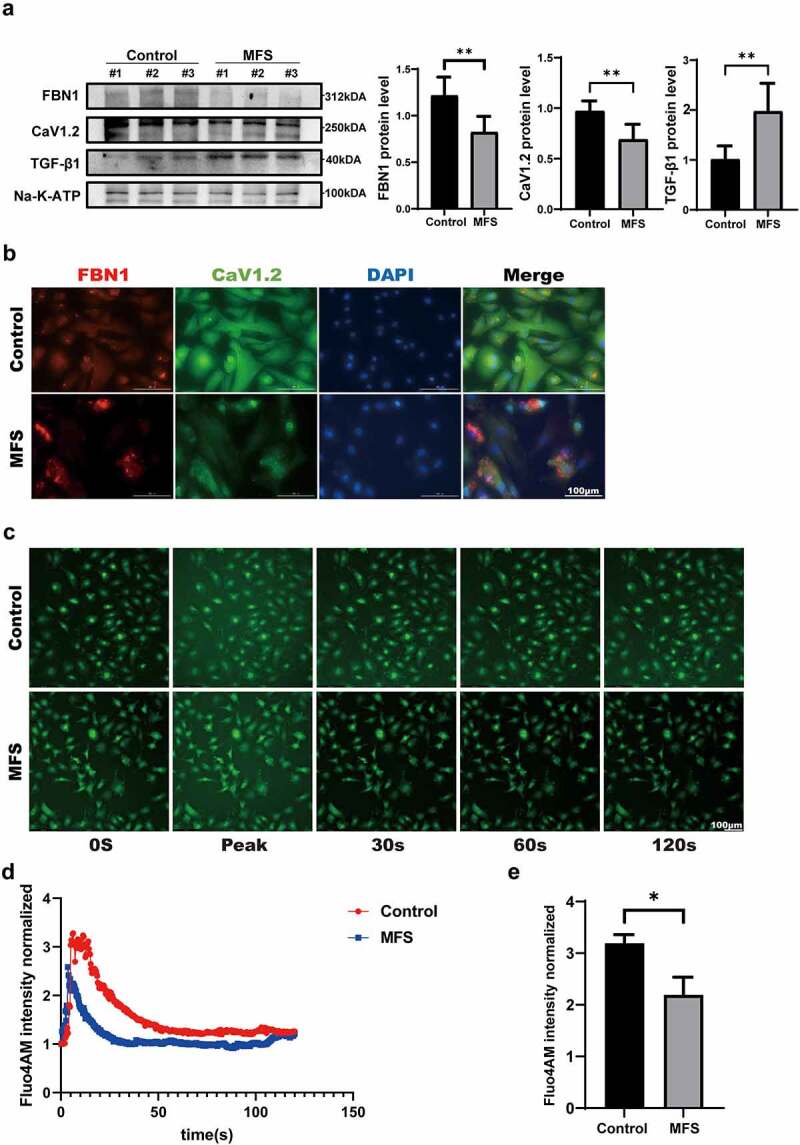
**The expression of Cav1.2 and intracellular calcium concentration is decreased by the reduction of FBN1 in MFS-HASMCs. (a)** Western blot and quantitative analysis of FBN1 and Cav1.2 protein levels in control-HASMCs and MFS-HASMCs. **(b)** Representative images of immunofluorescent staining for FBN1 and Cav1.2 in control-HASMCs and MFS-HASMCs. Scale bar = 100 μm. **(c)** Representative Fluo-4 Ca^2+^ images of control-HASMCs and MFS-HASMCs. Scale bar = 100 μm. **(d, e)** Determination of intracellular calcium concentration in control-HASMCs and MFS-HASMCs. Data are expressed as the mean ± SD. *p < 0.05, **p < 0.01. Source data are provided as a Source Data file.

### FBN1 deficiency inhibits the Cav1.2 expression and calcium influx in MFS-HASMCs

**Figure 3.**
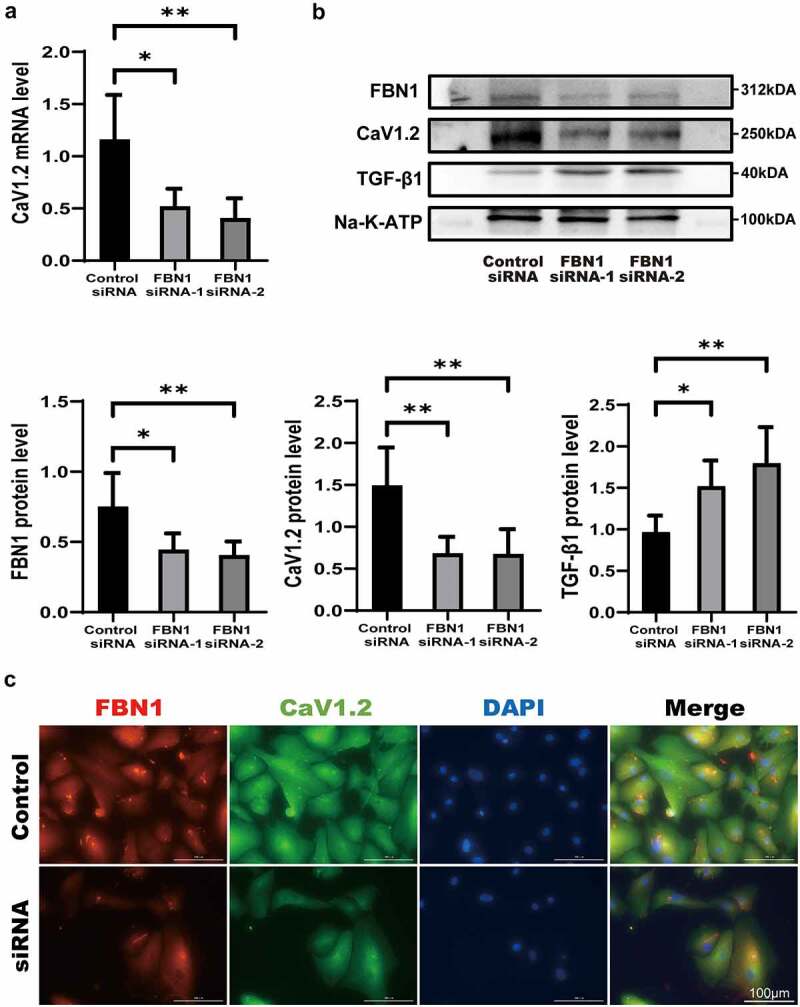
**Knockdown of FBN1 protein can reduce the expression of Cav1.2. (a)** RT-qPCR expression profile of Cav1.2 in control-HASMCs transfected with control-siRNA or FBN1-siRNA for 7d. **(b)** Western blot and quantitative analysis of FBN1 and Cav1.2 protein levels in control-HASMCs transfected with control-siRNA or FBN1-siRNA for 7d. **(c)** Representative images of immunofluorescent staining for FBN1 and Cav1.2 in control-HASMCs transfected with control-siRNA or FBN1-siRNA for 7d. Scale bar = 100 μm. Data are expressed as the mean ± SD. *p < 0.05, **p < 0.01. Source data are provided as a Source Data file.

Since mutations in FBN1 are the leading etiologies of MFS, we next investigated whether the decreased FBN1 expression found in MFS patients results in attenuation of the Cav1.2 expression. We first compared the protein levels of Cav1.2 between control-HASMCs and MFS-HASMCs. The expression of Cav1.2 was observed at a relatively lower level in MFS-HASMCs (Figure 2a). Additionally, immunofluorescence illustrated that FBN1 and Cav1.2 were at a lower level and were unevenly distributed in MFS-HASMCs (Figure 2b). To further explore the hypothesis that Cav1.2-mediated calcium influx was inhibited in MFS, the HASMCs were then preloaded with an intracellular calcium-sensitive fluorescent dye, Fluo-4AM. Inward currents were induced in HASMCs after exposure to 10 nM Cav1.2 agonist (Bay K8644). The results showed that the calcium influx rate and the maximal intracellular calcium concentration of MFS-HASMCs decreased, and the time course of intracellular calcium to return to normal remained unchanged (Figure 2, c-e). To further investigate if FBN1 regulates Cav1.2 expression, we used siRNA targeting FBN1 (siFBN1). FBN1 knockdown in HASMCs caused a significant reduction in Cav1.2 transcript levels (Figure 3a). The expression of Cav1.2 protein was also determined using both immunoblotting and immunofluorescence (Figure 3, b and c). These findings demonstrated that expression and physiological function of Cav1.2 are impaired by reducing FBN1 protein levels in MFS-HASMCs.

### FBN1 deficiency inhibits proliferation in MFS-HASMCs

**Figure 4.**
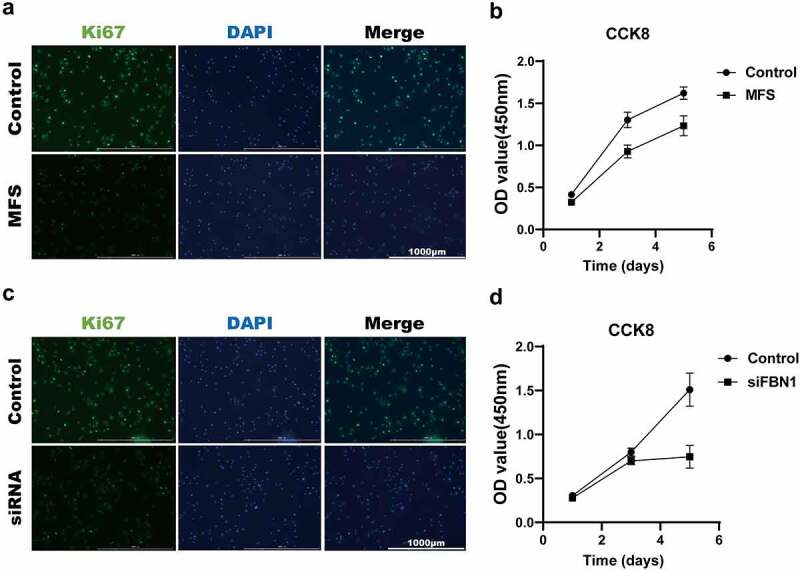
**FBN1 deficiency inhibits the proliferation of MFS-HASMCs. (a, b)** the proliferation levels of control-HASMCs and MFS-HASMCs were analyzed by immunofluorescence staining for Ki67 and CCK8 assay. Scale bar = 1000 μm.**(c, d)** the proliferation levels of control-HASMCs transfected with control-siRNA or FBN1-siRNA were analyzed by immunofluorescence staining for Ki67 and CCK8 assay. Scale bar = 1000 μm. Data are expressed as the mean ± SD. Source data are provided as a Source Data file.

Immunohistochemical staining of Ki67 showed that cell proliferation was lower in tissues from donors with MFS (Figure 1d). To examine whether FBN1 regulates cell proliferation, we conducted the following experiments. The cell proliferation levels of control-HASMCs and MFS-HASMCs were evaluated using immunofluorescence staining of Ki67 and CCK8 assay. MFS-HASMCs showed a lower level of cell proliferation on these tests (Figure 4, a and b). Consistent with these observations, a lower level of proliferation was observed in control-HASMCs transfected with siFBN1 than cells transfected with control siRNA (Figure 4, c and d). These findings demonstrated that cell proliferation is impaired by the reduction of FBN1 levels in MFS-HASMCs.

### TGF-β1 inhibits Cav1.2 expression

**Figure 5.**
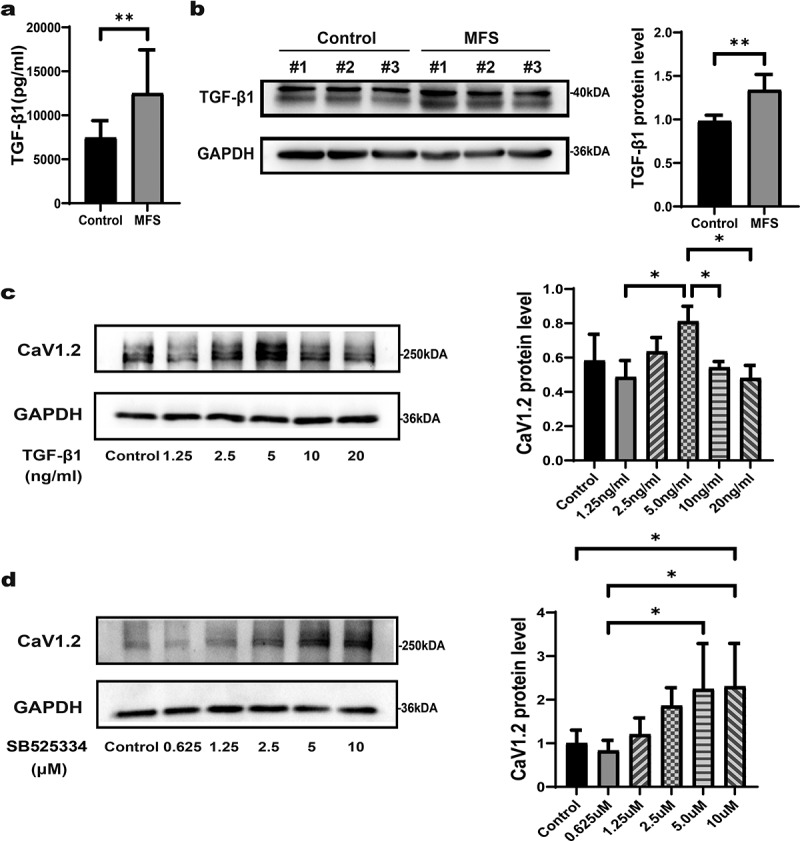
**The expression of Cav1.2 is inhibited by excessive TGF-β1 in MFS-HASMCs. (a)** Levels of TGF-β1 measured by ELISA in the serum of control donors and MFS patients. **(b)** Western blot and quantitative analysis of TGF-β1 protein levels in the ascending aorta of control donors and MFS patients. **(c)** Western blot and quantitative analysis of Cav1.2 in control cultures after treated with DMSO or TGF-β1 at 1.25, 2.5, 5, 10 or 20 ng/ml for 72 h. **(d)** Western blot and quantitative analysis of Cav1.2 in control cultures after treated with DMSO or SB525334 at 0.625, 1.25, 2.5, 5 or 10 μM for 72 h. Data are expressed as the mean ± SD. *p < 0.05, **p < 0.01. Source data are provided as a Source Data file.

TGF-β1 has been reported to be involved in the progression of MFS [22]. To investigate whether TGF-β1 can induce attenuation of Cav1.2 expression, we first measured TGF-β1 concentration in both the serum and aortic tissues from control donors and MFS patients. Compared with control donors, the levels of TGF-β1 were markedly upregulated in both the serum and aortic tissues from MFS patients (Figure 5, a and b). These data showed that TGF-β1 may participate in regulating Cav1.2 in MFS. To better understand the regulation of TGF-β1 on Cav1.2, control-HASMCs were treated with serial dilutions of TGF-β1. The data showed that TGF-β1 exerted concentration-dependent effects upon Cav1.2. Excessive concentration (>5 ng/ml) of TGF-β1 inhibited the expression of Cav1.2 (Figure 5c). To further confirm the correlation between TGF-β1 and Cav1.2, MFS-HASMCs were treated with various concentrations of TGFβRI inhibitor SB-525334 for 72 h. Results showed that 10 μM SB-525334 significantly increased Cav1.2 protein levels in a dose-dependent manner (Figure 5d). Taken together, the suppression of Cav1.2 expression is mediated by the over-expression of TGF-β1 in MFS-HASMCs.

### Proliferation levels of MFS-HASMCs are decreased by inhibition of Cav1.2

**Figure 6.**
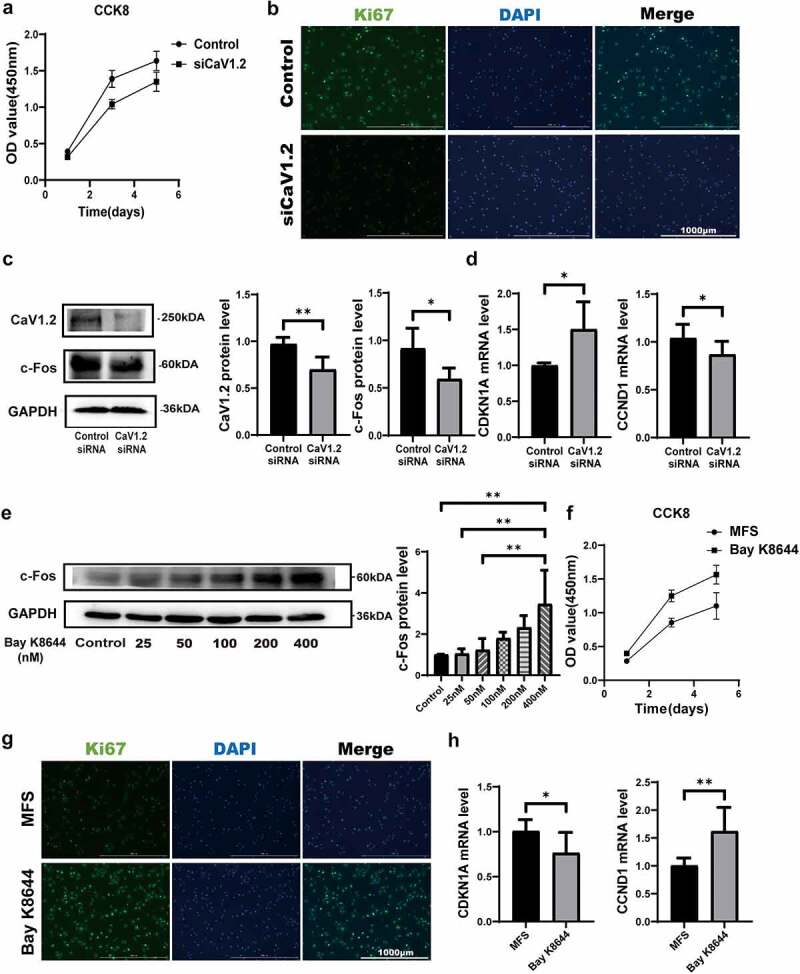
**Cell proliferation is regulated by the activity of Cav1.2. (a, b)** the proliferation levels of control-HASMCs transfected with control-siRNA or Cav1.2-siRNA were analyzed by CCK8 assay and immunofluorescence staining for Ki67, Scale bar = 1000 μm. **(c)** Western blot and quantitative analysis of Cav1.2 and c-Fos protein levels in control-HASMCs transfected with control-siRNA or Cav1.2-siRNA for 72 h. **(d)** RT-qPCR expression profile of CDKN1A and CCND1 in control-HASMCs after transfected with control-siRNA or Cav1.2-siRNA for 48 h. **(e)** Western blot and quantitative analysis of Cav1.2 in control cultures after treated with DMSO or Bay K8644 at 25, 50, 100, 200 or 400 nM for 72 h. **(f)** the proliferation levels of MFS-HASMCs were detected by CCK8 assay after cultured with 400 nM Bay K8644 for 72 h. **(g)** Immunofluorescence staining for Ki67 in MFS-HASMCs in control cultures after treated with DMSO or 400 nM Bay K8644 for 72 h. Scale bar = 1000 μm. **(h)** RT-qPCR expression profile of CDKN1A and CCND1 in MFS-HASMCs after treated with DMSO or 400 nM Bay K8644 for 48 h. Data are analyzed by two tailed Student’s t-test. Data are expressed as the mean ± SD. *p < 0.05, **p < 0.01. Source data are provided as a Source Data file.

As Cav1.2 plays crucial roles in regulating biochemical and electrical signaling of muscle cells [14], we hypothesized that the proliferation levels of MFS-HASMCs are decreased by inhibition of Cav1.2. To corroborate this hypothesis, control-HASMCs were transfected with control siRNA or Cav1.2 siRNA. After Cav1.2 knockdown, cell proliferation (Figure 6, a and b) and c-Fos protein levels (a transcription factor associated with cell proliferation) (Figure 6c) were reduced in control-HASMCs. Furthermore, we tested whether Cav1.2 knockdown could cause cell cycle arrest. Cav1.2 knockdown significantly promoted *CDKN1A* transcription but repressed *CCND1* transcription, leading to p53-dependent cell cycle arrest [23] (Figure 6d). Thus, the cell proliferation inhibited by reduction in Cav1.2 levels was at least partly dependent on c-Fos and *CDKN1A* activation. To test whether a Cav1.2 agonist (Bay K8644) would reverse decreased MFS-induced cell proliferation, MFS-HASMCs were treated with serial dilutions of Bay K8644 for 72 h. Protein levels of c-Fos peaked in MFS-HASMCs when cultured with 400 nM Bay K8644 (Figure 6e). Based on this result, MFS-HASMCs were cultured with 400 nM Bay K8644, and the levels of cell proliferation were detected by CCK8 assay and immunofluorescence staining of Ki67. Cell proliferation levels of MFS-HASMCs were significantly increased in the presence of Bay K8644 (Figure 6, f-h). These results demonstrate that the proliferation levels of MFS-HASMCs are decreased by inhibition of Cav1.2, and this pathological process can be reversed by a Cav1.2 agonist.

## Discussion

In the present study, we elucidated a novel FBN1/Cav1.2 pathway in MFS. For the first time, we found that the reduction in FBN1 levels suppresses expression of Cav1.2 under high concentration of TGF-β1. The Cav1.2 mediates Ca^2+^ influx into the cell and regulates a variety of cellular processes. Immunoblotting and immunofluorescence assays showed that Cav1.2 levels were lower in MFS-HASMCs, and the same phenomenon was observed in siFBN1-transfected cells. This is the first study to demonstrate a relationship between FBN1 and Cav1.2. It has been accepted that the sustained elevation of cytosolic Ca^2+^ concentration mainly depend on the influx of extracellular Ca^2+^ by Cav1.2 [14]. Cytosolic Ca^2+^ participates in various physiological processes by regulating calmodulin and calcineurin. Previous studies have demonstrated that calmodulin antagonists trigger G1 phase cell cycle arrest [24] and calcineurin is required for the expression of cyclin D1 [25]. NFATc1, activated by calcineurin, delays cell senescence by downregulating cell cycle inhibitor p21 [26]. A decrease in intracellular calcium ions will decrease proliferation and increase apoptosis. In our results, The result showed that the Cav1.2 protein level of Marfan cells decreased (Figure 2b), and the level of cell proliferation decreased (Figure 3, a and b). In the process of primary cell culture, the arrangement of MFS- HASMCs was looser, the maximum cell density was lower, and the more dead cells could be observed in MFS-HASMCs compared to NC-HASMCs. The pathological changes of MFS-HASMCs are consistent with the changes caused by the decrease of calcium influx. The pathological process of MFS may be mediated by the inhibition of Cav1.2. Notably, Cav1.2 can be used as a target to explore the regulatory mechanisms of MFS.

TGF-β1 was previously reported to inhibit Cav1.2 expression in cortical neurons [27]. Our study found that high concentration TGF-β1 inhibited Cav1.2 expression and cell proliferation. TGF-β1 inhibitor SB-525334 reversed the change in MFS-HASMCs, but excessive increase of inhibitor had adverse effects. Mounting evidence supports the view that the suitable concentration of TGF-β1 is involved in the stability and maintenance of the vascular. A suitable extracellular concentration of TGF-β1 is reported to promote endothelial cells (ECs) proliferation, whereas abnormally high or low extracellular concentration of TGF-β1 lead to cytostasis [28]. Losartan has been shown to inhibit aortic dilation by inhibiting the expression of TGF-β ligand and receptor in MFS [29,30]. And low levels of TGF-β1 can also exacerbate the progression of aneurysms. TGF-β1 neutralizing antibodies [31] or *TGFBR2* mutations can increase the instability of aneurysms [32]. Taken together, extracellular TGF-β1 concentration will enable the development of new therapeutic interventions for modulating vascular proliferation. This may be a novel strategy to delay aneurysm formation and prevent aneurysm rupture.

VSMCs are attracted to abnormal areas when blood vessels are injured or diseased. VSMCs proliferate and synthesize specific proteins or other molecules to promote the repair of blood vessels and prevent the progression of disease [33]. In the blood vessels of patients with MFS, the proliferation activity of HASMCs is decreased. Therefore, the aortic root in Marfan patients is less likely to repair damage. The progression of MFS can be delayed by Bay K8644 via reversing the inhibition of cell proliferation. Furthermore, patients with MFS often present muscle hypotonia and visual problems. It is reported that Bay K8644 significantly enhanced the maximum amplitude of the frog’s twitch response [34]. LTCCs are essential for the development and maintenance of basal tone in porcine retinal veins [35]. The above findings suggest that Cav1.2 agonist can serve as a standalone agent for the treatment of MFS by rescuing the proliferation of MFS-HASMCs.

The proliferation markers used in this study were limited, and the signaling pathways between TGF-β1 and Cav1.2 need to be further studied. Further, the effect of Bay K8644 was detected only on HASMCs, but should additionally be tested on other cell types, such as skeletal muscle cells, optic nerve cells and vascular endothelium cells. We have only demonstrated the relationship between Cav1.2 and cell proliferation in MFS patients. In the future, we hope to examine the roles of Cav1.2 in cellular contractility, calcification, and inflammation in MFS patients.

## Conclusions

Collectively, the study is the first to elucidate the FBN1 deficiency decreases the expression levels of Cav1.2 by regulating TGF-β1, and the downregulation of Cav1.2 inhibits cell proliferation of MFS-HASMCs. Cav1.2 represents a novel therapeutic target for the treatment of MFS.

## Supplementary Material

Supplemental Material

## Data Availability

The data that support the findings of this study are available from the corresponding author, Buqing Ni, upon reasonable request.
