# Supplementary material for: Fibrillin-1 mutation contributes to Marfan syndrome by inhibiting Cav1.2-mediated cell proliferation in vascular smooth muscle cells
Source: Channels (Austin). 2023 Mar 27;17(1):2192377. doi: 10.1080/19336950.2023.2192377 (PMC10054150; doi:10.1080/19336950.2023.2192377)
Supplement: Supplemental Material [file KCHL_A_2192377_SM7575.docx]

**
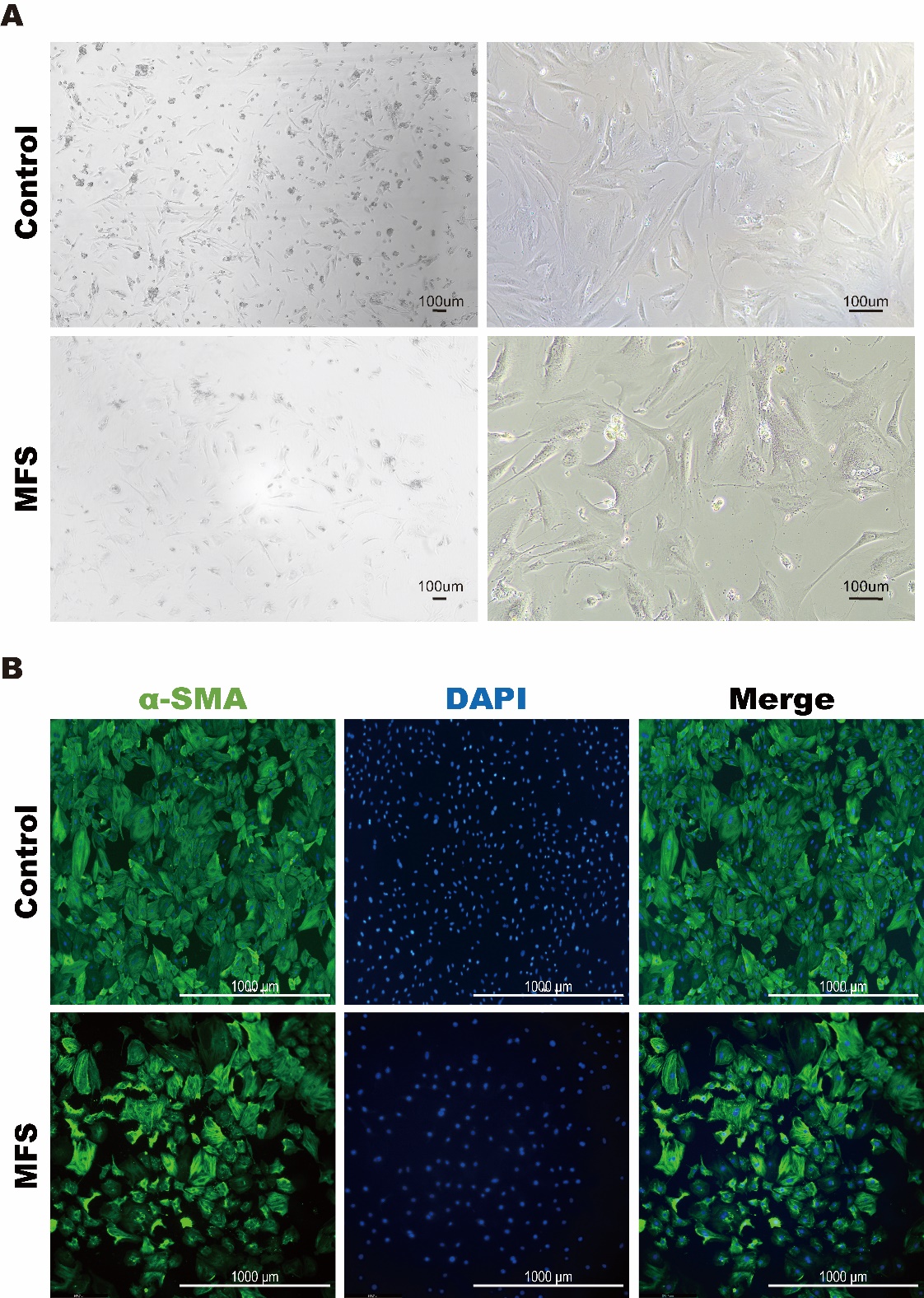
Supplemental Figure 1**

**A.** Cell morphology observed under a microscope. **B.** Representative images of immunofluorescent staining for α-SMA in control-HASMCs and MFS-HASMCs. Scale bar=1000 μm.

**
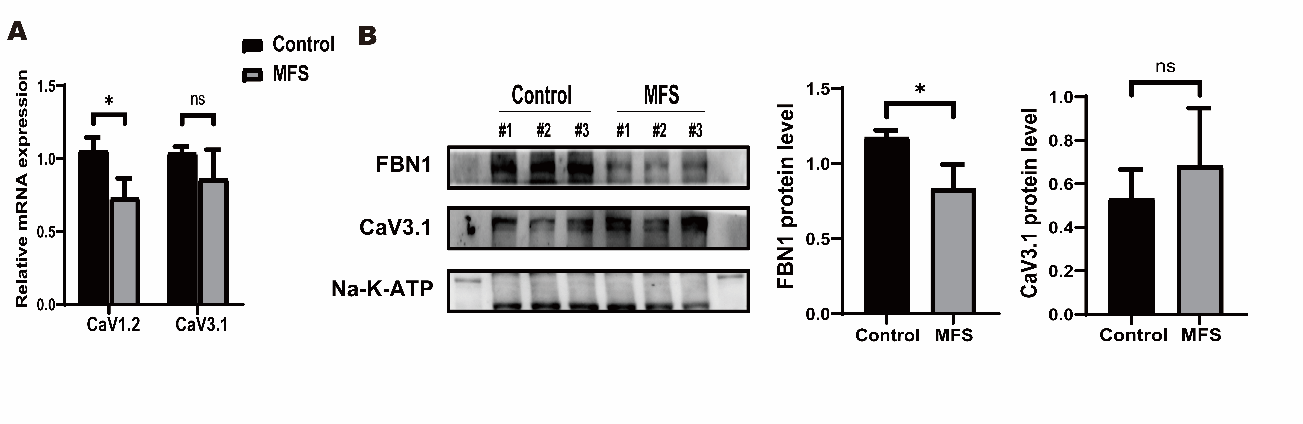
**

**Supplemental Figure 2**

**A. RT-qPCR expression profile of Cav1.2 and Cav3.1 in the ascending aorta of control donors and MFS patients. B. Western blot and quantitative analysis of FBN1 and Cav3.1 protein levels in the ascending aorta of control donors and MFS patients.**

**Supplemental Table 1: The antibodies used in this study**

| **Anti-Cav1.2 (CACNA1C)** | **Alomone Labs (Israel, Jerusalem) # ACC-003** | **WB: 1:1000; IF: 1:50** |
| --- | --- | --- |
| **Anti-FBN1** | **Abcam (Cambridge, UK) #ab53076** | **WB: 1:500** |
| **Anti-FBN1** | **Thermo Fisher (Massachusetts, United States) # MA5-12770** | **IF: 1:50** |
| **Anti-c-Fos** | **Proteintech Group, Inc (Chicago, United States)**  **# 66590-1-Ig** | **WB: 1:500** |
| **Anti-GAPDH** | **Proteintech Group, Inc (Chicago, United States)**  **#60004-1-Ig** | **WB: 1:10000** |
| **Anti-Ki67** | **Proteintech Group, Inc (Chicago, United States)**  **#27309-1-AP** | **IF: 1:250; IH: 1:250** |
| **Anti-rabbit** | **ZSGB-Bio (Beijing, China) #ZB-2301** | **WB: 1:7500** |
| **Anti-mouse** | **ZSGB-Bio (Beijing, China) #ZB-2305** | **WB: 1:7500** |
| **Anti-Na+/K+-ATPase α1** | **Bioworld Technology,Inc (Minnesota, United States) #BS1436** | **WB: 1:1000** |
| **Anti-rabbit- Alexa Fluor 488** | **Servicebio (Wuhan,China) #GB25303** | **IF: 1:250** |
| **Anti-mouse- Alexa Fluor Cy3** | **Servicebio (Wuhan,China) #GB21401** | **IF: 1:250** |

**Supplemental Table 2: The Primers of Relative Genes in this study**

| **Gene name** | **Primers** |
| --- | --- |
| **GAPDH Forward (5’- 3’)** | **CATGTTCGTCATGGGTGTGAACCA** |
| **GAPDH Reverse (5’- 3’)** | **AGTGATGGCATGGACTGTGGTCAT** |
| **FBN1 Forward (5’- 3’)** | **GGTGAATGTACAAACACAGTCAGCA** |
| **FBN1 Reverse (5’- 3’)** | **ATAGGAACAGAGCACAGCTTGTTGA** |
| **CACNAC1C Forward (5’- 3’)** | **TTGAGCAACCTTGTGGCATCCTTG** |
| **CACNAC1C Reverse (5’- 3’)** | **ACGGGTCTGCATCTCATCGAAGTT** |
| **TGF-β1 Forward (5’- 3’)** | **GGCCAGATCCTGTCCAAGC** |
| **TGF-β1 Reverse (5’- 3’)** | **GTGGGTTTCCACCATTAGCAC** |
| **CCND1 Forward (5’- 3’)** | **CCCGCACGATTTCATTGAAC** |
| **CCND1 Reverse (5’- 3’)** | **AGGGCGGATTGGAAATGAAC** |
| **CDKN1A Forward (5’- 3’)** | **TGGAGACTCTCAGGGTCGAAA** |
| **CDKN1A Reverse (5’- 3’)** | **GGCGTTTGGAGTGGTAGAAATC** |

**Supplemental Table 3: The Relative siRNA in this study**

| **Gene name** | **Relative sequences (5’ - 3’)** |
| --- | --- |
| **FBN1 siRNA-1** | **GTCCAATGATGACATGTGA** |
| **FBN1 siRNA-2** | **GAAAGAACTTAACCAACTA** |
| **Cav1.2 siRNA** | **CAGGGATGTTAGTCTGTAT** |

**Supplemental** **Table 4: Transcriptomic analysis of voltage-gated calcium channel (P<0.05)**

| **ID** | **adj.P.Val** | **P.Value** | **t** | **logFC** | **Gene.symbol** | **Gene.title** | **Gene.ID** |
| --- | --- | --- | --- | --- | --- | --- | --- |
| **ILMN_1673503** | **0.9931** | **1.26E-02** | **-3.18458** | **-1.337822** | **CACNB4** | **calcium voltage-gated channel auxiliary subunit beta 4** | **785** |
| **ILMN_1861819** | **0.9931** | **3.16E-02** | **-2.59158** | **-0.9902287** | **CACNA1C** | **calcium voltage-gated channel subunit alpha1 C** | **775** |
| **ILMN_1711049** | **0.9931** | **3.37E-02** | **2.550043** | **1.3313519** | **CACNA2D2** | **calcium voltage-gated channel auxiliary subunit alpha2delta 2** | **9254** |
| **ILMN_1703016** | **0.9931** | **4.08E-02** | **2.429209** | **0.7723134** | **CACNA1G** | **calcium voltage-gated channel subunit alpha1 G** | **8913** |
| **ILMN_1724306** | **0.9931** | **4.78E-02** | **2.328466** | **1.0902443** | **CACNG1** | **calcium voltage-gated channel auxiliary subunit gamma 1** | **786** |
